# Citizen science applied to building healthier community environments: advancing the field through shared construct and measurement development

**DOI:** 10.1186/s12966-017-0588-6

**Published:** 2017-09-29

**Authors:** Erica Hinckson, Margaret Schneider, Sandra J. Winter, Emily Stone, Milo Puhan, Afroditi Stathi, Michelle M. Porter, Paul A. Gardiner, Daniela Lopes dos Santos, Andrea Wolff, Abby C. King

**Affiliations:** 1 Auckland University of Technology, Faculty of Health and Environmental Sciences, National Institute of Public and Mental Health, Centre for Child Health Research Centre for Active Ageing, Private Bag, 92006 Auckland, New Zealand; 2 Department of Planning, Policy and Design, School of Social Ecology, University of California, Irvine, Irvine, CA USA; 3 Stanford Prevention Research Center, Department of Medicine, Stanford University School of Medicine, Stanford, CA USA; 4 University of Zurich, Epidemiology, Biostatistics and Prevention Institute, Zurich, Switzerland; 5 University of Bath, Bath, UK; 6 University of Manitoba, Faculty of Kinesiology and Recreation Management, Centre on Aging, Winnipeg, Canada; 7 The University of Queensland, Faculty of Medicine, Brisbane, Australia; 8 Federal University of Santa Maria, Santa Maria, Brazil; 9 Friedrich-Alexander University Erlangen, Institute of Sport Science and Sport (ISS), Nuremberg, Germany; 10 Division Of Epidemiology, Department Of Health Research & Policy, Stanford University School Of Medicine, Stanford, CA USA

**Keywords:** Community, Constructs, Measures, Our voice, Residents, Stanford healthy neighborhood discovery tool

## Abstract

**Background:**

Physical inactivity across the lifespan remains a public health issue for many developed countries. Inactivity has contributed considerably to the pervasiveness of lifestyle diseases. Government, national and local agencies and organizations have been unable to systematically, and in a coordinated way, translate behavioral research into practice that makes a difference at a population level. One approach for mobilizing multi-level efforts to improve the environment for physical activity is to engage in a process of citizen science. Citizen Science here is defined as a participatory research approach involving members of the public working closely with research investigators to initiate and advance scientific research projects. However, there are no common measures or protocols to guide citizen science research at the local community setting.

**Objectives:**

We describe overarching categories of constructs that can be considered when designing citizen science projects expected to yield multi-level interventions, and provide an example of the citizen science approach to promoting PA. We also recommend potential measures across different levels of impact.

**Discussion:**

Encouraging some consistency in measurement across studies will potentially accelerate the efficiency with which citizen science participatory research provides new insights into and solutions to the behaviorally-based public health issues that drive most of morbidity and mortality. The measures described in this paper abide by four fundamental principles specifically selected for inclusion in citizen science projects: feasibility, accuracy, propriety, and utility. The choice of measures will take into account the potential resources available for outcome and process evaluation. Our intent is to emphasize the importance for all citizen science participatory projects to follow an evidence-based approach and ensure that they incorporate an appropriate assessment protocol.

**Conclusions:**

We provided the rationale for and a list of contextual factors along with specific examples of measures to encourage consistency among studies that plan to use a citizen science participatory approach. The potential of this approach to promote health and wellbeing in communities is high and we hope that we have provided the tools needed to optimally promote synergistic gains in knowledge across a range of Citizen Science participatory projects.

## Background

Physical inactivity across the lifespan remains a public health issue for many developed countries [1]. Physical inactivity has contributed to the pervasiveness of lifestyle diseases and conditions (e.g., cardiovascular disease, diabetes, obesity) [2]. There is a significant body of evidence that supports behavioral strategies to increase physical activity [3]. However, government, national and local agencies and organizations have been unable to systematically, and in a coordinated way, translate behavioral research into practice to increase physical activity at a population level.

The built environments where people live, work, receive education, socialise, engage in recreation, and access destinations (services, retail, nature) can affect physical activity levels [4–9]. One effective approach for increasing a community’s physical activity levels is to link multiple sectors (i.e., the individual, the community, local and regional government) in efforts to create health-promoting local environments and policies. Clinical and population scientists know the many short- and long-term benefits of physical activity across the population [10]. A critical next step is to target structural changes to local environments to support individuals’ regular physical activity irrespective of age, socioeconomic status, culture, or ethnicity [11].

Citizen science, a concept utilised originally by the disciplines of ecology and environmental sciences, is one promising approach for bringing together multiple sectors to address the physical activity environment. “Citizen Science” is typically defined as an approach involving members of the public working closely with research investigators to initiate and advance scientific research projects [12]. It capitalises on people’s innate sense of curiosity as well as desire to learn, question, contribute, and interact with others around issues and areas that impact a community’s health and/or well-being. Note that while the term “citizen science” is the most commonly used descriptor in this field, we use this term to broadly denote community resident participation irrespective of formal citizenship status.

Community-Based Participatory Research (CBPR), is an approach in which involved participants are directly involved in and affected by the research questions being addressed [13, 14]. In a comprehensive CBPR process, it is typically recommended that community members be included in the following four ways: (1) they participate as partners rather than study subjects; (2) they contribute their knowledge to understanding and addressing health problems; (3) they often contribute to the research process through participation in the conception and design of the project, data collection, or/and participation in data analysis; and (4) they typically receive immediate benefits from the research results in their community [15].

The *Our Voice* citizen science model seeks to build on and combine both of these perspectives in the following manner: 1) it brings a systematic methodology (via use of the Discovery Tool app) to resident-based “real-world” data collection that is a hallmark of scientifically-oriented citizen science methods but often lacking in many CBPR processes; and 2) it systematically involves residents in data insight generation, consensus building, and data-based local community applications that are typically missing from the citizen science field.

Taken together, the *Our Voice* citizen science participatory approach empowers residents not only to systematically collect signficant and meaningful local information about their community environments, but also to participate by prioritising their concerns and their own interpretations of the data in order to engage meaningfully in cross-sector conversations to generate practical solutions that can have direct impact on their own community. A citizen science participatory approach has the potential to enable the following outcomes: i) multi-sector and intergenerational approaches for sustainable local change; ii) greater equality, where disadvantaged groups can become key stakeholders in the local discovery and change process; and iii) realistic and contextualized solution(s), as opposed to a “one size fits all” approach to local change. This approach also employs an economy of resources by tapping into existing human resources, knowledge, and networks “in situ”. Additionally, this approach enables direct translation and application of findings to those who will benefit the most directly. As well as being a useful method of data collection, citizen science can enhance public understanding of science, and strengthen links between professional scientists and community members [16].

As citizen science participatory research gains momentum, scientific synergies can be realized by identifying common constructs (i.e., theoretical concepts) that should be measured to document and evaluate the process. In this paper, we describe overarching categories of constructs that should be considered when designing citizen science participatory projects expected to yield multi-level interventions for physical activity. We also provide an example of a citizen science participatory approach to promoting physical activity. Where possible, this paper also suggests available tools to assess some of the core constructs that are described. Owing to the complexity of a number of the concepts, however, and the relevance of cultural contexts, these suggested measures will not be universally suited to all studies. While sharing commonalities, each project is expected to address unique behaviors and/or environmental features, the variety of which typically cannot be anticipated a priori*.*

In offering a list of relevant constructs together with a set of candidate measurement tools, we seek to stimulate researchers utilizing the citizen science participatory approach to engage in a thoughtful process of identifying relevant, useful, and feasible measures that may be incorporated into a particular study and compared across multiple studies. It is incumbent on the researcher to select the measures that fit best within the population, setting, and theoretical foundations of a given citizen science project. New measures, formulated for use in specific populations, are being developed continuously, so we do not purport to present a comprehensive or authoritative list. It is assumed that researchers will be familiar with the basic principles of evaluation research design. The goal of this paper is to outline a set of constructs that cut across many settings and theoretical models, and recommend that the architects of each citizen science project identify which constructs and associated measures can and should be assessed within each study.

Based on the above considerations, the purpose of this paper is to recommend common constructs and potential measures across different levels of impact (i.e., individual, social, built environment, community, policy) for citizen science participatory research targeting physical activity. By encouraging consistency in measurement constructs across multiple studies, we intend to accelerate the pace at which individual scientific gains will build on one another to yield high-quality evidence to inform intervention guidelines. We believe that such coordination will magnify the impact of each individual project and lead to greater forward strides in promoting community-based health and well-being than might be accomplished by a multitude of isolated, uncoordinated studies. It should be noted that while this is not an exhaustive list, it provides examples of the types of constructs across different levels of impact that have been found to be relevant in prior citizen science participatory research in the physical activity area [12].

## Constructs

The multiple levels of constructs that may be affected by or may influence a citizen science participatory process for improving physical activity include individual, interpersonal, environmental and policy [17]. In addition, the content of the intervention should be documented using a process evaluation. The following section describes constructs recommended for consideration in selecting measures to be included in a citizen science physical activity intervention study. Specific examples of measures to assess these constructs are presented subsequently.

### Individual-level constructs

#### Demographic characteristics

At the individual level, social and population health scientists typically collect, at a minimum, information about the age and sex of research participants. In relation to health behaviors, there is ample evidence that both age and sex correlate with physical activity [18]. Another individual-level characteristic that has been shown to have consistent associations with physical activity is socioeconomic status (SES) which typically includes level of education and/or household income [19–22]. It is recommended that some indicator of SES also be included. Other frequently measured demographic characteristics include marital status and number of people in the household-variables which reflect potentially social factors that can impact intervention effectiveness [23]. Health status, whether it is self-rated health or reported as number of morbidities, is another important demographic especially for older adults [24].

#### Advocacy skills

Advocacy means taking action on behalf of oneself or others. In a citizen science project, advocacy skills are critical to ensure that concerns within the community are raised effectively to local councils or boards. While there are many types and levels of advocacy (e.g., self, peer, group, systems and legal), [25–27], here we are referring to the minimum level of personal skills typically needed to engage in group advocacy.

#### Empowerment

Empowerment is the process of becoming more confident to take action for the improvement of one’s life or the local context in which one lives. The literature in this area often has focused on empowerment as seen in patient engagement in self-management [28–30], in a variety of professions (e.g., nursing, teaching) [31, 32] and among consumers of services [33, 34].

#### Civic engagement

Civic engagement is the meaningful participation of residents in building and maintaining a community [35] or actively participating in the life of their community through activities such as voting, joining community groups, and volunteering [36]. It has been argued that civic engagement is not simply the act of volunteerism but also includes other activities associated with civic life, including active involvement in the community, staying informed about current affairs, and making informal connections with other residents, as well as community organizations, around issues that impact multiple residents [37].

#### Personal self-efficacy

A wealth of research supports the role of self-efficacy (a person’s level of confidence in his or her ability to engage in a specific behavior in a given context) as a determinant of behavior [38]. Moreover, self-efficacy can be nurtured through a variety of strategies, including modeling, shaping, and mastery experiences [38]. In the context of citizen science interventions to promote community health and well-being, program success may be shaped by and may result in changes in residents’ self-efficacy related to a variety of behaviors.

#### Health behavior

The primary focus of this paper is on resident-engaged interventions to promote community health and well-being through increasing physical activity. As part of specific interventions that target physical activity, the type of physical activity that is expected to change should be carefully considered. Possibilities include recreational activity, structured forms of exercise, active transport, and decreased sedentary time.

### Interpersonal-level constructs

#### Social norms

Social norms are important determinants of health behaviors [39], and have been incorporated into a number of health behavior theories. Assessment of social norms may assist in the interpretation of findings, especially within cultures that may discourage physical activity among certain subgroups of the population [40].

#### Neighborhood cohesion

When residents characterize a neighborhood as having strong social bonds and mutual trust in the absence of social conflict, that neighborhood may be described as having high cohesion [41]. A growing number of studies support the assertion that neighborhood cohesion influences residents’ health behaviors [42].

### Environment-level constructs

#### Urban/rural

Although there are numerous definitions, urban areas tend to be characterized based on population density, degree of urbanization and/or proximity to a metropolitan area [43]. Urban environments have been found to offer a different set of opportunities and hindrances for healthy behaviors as compared to suburban or rural environments [44–46].

#### Poverty

In addition to the impact of individual-level socioeconomic status on health behavior, there appears to be an additional influence of community-level poverty on such behaviors [47]. Community-level indicators of poverty may indicate reduced access to retail outlets selling fresh produce, limited recreational facilities, and elevated crime rates, all features of an obesogenic environment [48].

#### Safety

Although it seems intuitive that neighborhood safety will influence the likelihood of engaging in regular physical activity, a recent review of the literature [49] did not find support for this direct association. The authors pointed out several study limitations and suggested that a number of moderators need to be taken into account in future studies (e.g., age, sex, motivation to be physically active) in future studies. They call for additional studies that examine the longitudinal association between residents’ safety and their participation in physical activity as well as studies that use valid measures and incorporate multi-level models.

#### Weather, air quality and excessive noise

Outdoor temperature and air quality are atmospheric environmental factors that may impact the safety or acceptability of outdoor physical activity [50]. Extremes of low or high temperatures or conditions that compromise outdoor safety (e.g., sidewalks covered in snow and ice) [51] may prevent physical activity participation throughout some seasons. Winter conditions often create barriers for community mobility particularly in older adults [52]. Likewise, public health officials recommend that individuals avoid outdoor activities when thresholds are exceeded for various air pollutants. This is particularly true for those with breathing or heart conditions, because of the potential to trigger adverse health events (e.g., asthma attacks; [53]). However, recent research shows that the benefits of physical activity usually outweigh the costs of pollution [54]. While less rigorously studied in the physical activity field, there are growing concerns about the potential impacts of excessive environmental noise levels on outdoor physical activity participation levels among certain population subgroups, including aging adults [55].

#### Walkability

Walkability refers to the extent to which an area or neighborhood is friendly to walking. It has been suggested that neighborhoods with greater mixed land use, street connectivity, and residential density [56] are more “walkable” for many different groups of residents. Recent research has identified four environmental attributes that were positively and linearly related to physical activity in 14 cities worldwide, which include the following factors: net residential density, intersection density, public transport density, and number of parks [11]. It was also reported that the difference in physical activity between participants living in the most and least walkable neighborhoods ranged from 68 to 89 min/week, representing up to 60% of the recommended physical activity guidelines [11]. With respect to children’s physical activity, density and accessibility have been positively associated with walking to school [57] while distance to school has been negatively associated with sedentary behavior [8].

#### Public transport

Much of the literature regarding access to public transport is derived from city planning [58], and statistical modelling [59], or exploration and analysis related to specific locales or settings (e.g., station terminals) [60]. A review of 27 studies examining the link between use of public transport and physical activity found that the median walking time associated with use of public transport was 15 min from a public transport stop [61]. Moreover, living in a community in the highest 5% of public transportation density was associated with a mean weekly accumulation of moderate-to-vigorous activity (MVPA) roughly 30 min higher as compared to living in a community in the lowest 5% of public transportation density [11].

### Policy-level constructs

Policies relevant to community-based health promotion efforts may include courses of action adopted by local housing associations or neighborhood groups, as well as civic government at all levels. Common policies that may impact residents’ likelihood of engaging in physical activity can include allocation of resources (e.g., a municipality directing resources to enhance outdoor recreation areas) and changes in zoning regulations (e.g., designation of areas for mixed use of both residential and retail). Moreover, residents’ willingness to engage in the citizen-science process may be facilitated by policies that encourage the exchange of information with local government, such as the establishment of neighborhood councils with direct representation to the city or area council.

### Process evaluation

Replication of any intervention study requires thorough documentation of the key elements of the intervention, often described as process evaluation. A comprehensive process evaluation ensures that the program’s operations, implementation, and service delivery are thoroughly documented. Process data may also be used to help interpret study findings and identify program elements that were more or less effective [62]. In this process, it is important to collect data to show whether the program was implemented as desired [63]. Some examples include the following: identification of strategies used to recruit community partners and citizen scientists, response rates and reasons for non-response (if available), neighbourhood selection criteria, type and amount of citizen scientist training delivered, process by which themes were identified and prioritized by the citizen scientists, process followed regarding the identification and listing of the solutions generated by the citizen scientists, number of meetings with local and public stakeholders, documenting meeting agendas and lists of attendees from meetings with stakeholders, noting participants’ views in addition to likes and dislikes as part of the community meetings, reporting the number of people reached during project implementation (e.g., count of attendance in meetings, which stakeholders joined the meetings, etc.), and time and type of researchers, citizen scientists, and other community organizations or facilitators who were involved. These data may be collected by researchers with the assistance of citizen scientists, and/or by participating organizations serving as partners with researchers. The data are usually collected during and immediately after project implementation. A variety of methods can be used including counts or other types of observational assessments, and focus group and/or interview-derived transcription. Clear documentation of individual citizen science interventions will facilitate comparisons among studies and synergistic gains in knowledge.

## Measures

Examples of measures that may be used to assess the constructs described above are presented in Table 1. Measures should be chosen, to the extent possible, according to their adherence to the following four principles (adapted from the Joint Committee on Standards for Educational Evaluation [64]). These are principles that novice and experienced researchers are encouraged to consider:

- Feasibility (ease of data collection). Feasibility standards ensure that the evaluation is viable and pragmatic, and emphasize that the evaluation should employ practical, non-disruptive procedures; that the differing political interests of those involved should be anticipated and acknowledged; and that the use of resources in conducting the evaluation should be prudent and produce valuable findings.
- Accuracy (focusing on sensitivity to change). The use of an established instrument with appropriate validity and reliability is preferred. For areas in which an instrument needs to be developed (e.g., in relation to specific populations or subgroups, or to measure constructs that are reasonably new to the field), evaluation of test-retest reliability and other psychometric properties is encouraged. For example, test-retest reliability can often be ascertained through use of a control or comparison group that is not receiving the intervention. Moreover, the tool must be able to reliably detect and capture the small changes that one might realistically expect from a community-based citizen science intervention.
- Propriety (conformity to conventionally accepted standards of behavior). Propriety standards ensure that the evaluation is ethical (i.e., conducted with regard for the rights and interests of those involved and effected), and address such items as developing protocols and other agreements for guiding the evaluation; protecting the welfare of human participants; weighing and disclosing findings in a complete and balanced fashion; and addressing any conflicts of interest in an open and fair manner.
- Utility (usefulness, of benefit). Data collected from the assessment instrument should be easy to interpret, avoiding complicated data cleaning or preliminary analysis to produce a result.

**Table 1:** Examples of measures for assessing constructs

Construct	Measures
Individual	
Demographics	Sex^a^
	Age/Date of Birth^a^
	Socioeconomic Status: Educational attainment^a^
	Number of people in household^a^
	Health status^a^
Advocacy Skills	Social Issues Advocacy Scale (SIAS) [75] ^a^
Empowerment	Personal Empowerment Scale [76]^a^
Civic Engagement	Volunteer experience [77]^a^
	Civic engagement scale (adolescents) [78]^a^
Self-efficacy	The Older Adults’ Computer Technology Attitudes Scale [52]^a^
	Computer Use Self Efficacy Scale [53]^a, d^
	Self-efficacy for Exercise Scale [79]^a^
Health Behavior	Community Healthy Activities Model Program for Seniors (CHAMPS) questionnaire for midlife and older adults [80]^a^
	International Physical Activity Questionnaire version for ages 15–65 years (IPAQ; 54) [81]^a^
	Accelerometry & related device-based assessment tools [82]^a^
Interpersonal	
Social Norms	Injunctive Norms [83]^a^
Neighborhood Cohesion	Neighborhood cohesion [84]^a^
	Social Network Analysis [28]^b^
Environment	
Urban/Rural	e.g., U.S. Centers for Disease Control and Prevention classification of counties (https://www.cdc.gov/nchs/data_access/urban_rural.htm)^b^
Poverty	e.g., English Indices of Deprivation (https://www.gov.uk/government/statistics/english-indices-of-deprivation-2015)^b^
Safety	Perceived neighborhood safety ([85])^a^
	Neighborhood Incivilities From the Neighborhood Inventory of Environmental Typology (NlfETy) [86]^b^
Weather/Air Quality	Ambient temperatures^b^
	Ambient fine particulate matter^b^
	Historical weather data2 (e.g., Environment Canada: http://climate.weather.gc.ca/index_e.html) ^b^
Walkability	Abbreviated Neighborhood Environment Walkability Scale; ANEWS [59]^a^
Public Transport	e.g., Walking distance to a transit stop/station from residential address is less than 400 m for bus stop or train or less than 800 m to train/rail station access [58]
Policy	
Allocation of Resources	New funds allocated to environmental resources to support PA^c^
Zoning	Number of changes to zoning ordinances designed to promote environmental support for PA^c^
Community Engagement in Government	Proportion of residents actively participating in local government meetings^c^
	Office-holders’ perceived level of impact that residents have on local government decisions^c^
Process Evaluation	
Intervention components	Fidelity of intervention^c^
Acceptability of intervention components	Satisfaction with the intervention^a,c^
Intervention Reach	Awareness of the intervention^a,c^

^a^Assessed at the level of the individual

^b^Assessed in the aggregate or via existing databases

^c^Assessed via document review, observation, and/or key informant interviews

^d^Increasingly, information technology is being used to facilitate citizen science projects [87]. For citizen science projects that incorporate this type of technology, measuring individual level self-efficacy at baseline can inform the amount of technology support that participants may require, and measuring this construct at follow up may indicate how participation in an information technology-driven citizen science project has changed the confidence with which participants interact with technology

## Example: Our voice global citizen science engagement and advocacy framework

To provide an example of how the constructs and measures outlined above can be implemented to improve physical activity, the Our Voice citizen science example of King and colleagues [12] will be described. While physical activity and increased walkability have been a focus of a number of Our Voice studies to date, the Our Voice framework is being applied to a number of other health areas as well, including healthy food access, reduction of environmental stressors, and violence prevention. The Our Voice Citizen Science Engagement and Advocacy Framework (i.e., the Our Voice Framework) describes a process in which community residents are trained as “citizen scientists” who not only collect relevant data about their local contexts and environments, but also become agents of positive health-enabling changes in their communities through community assessment, engagement, advocacy, and action [12]. Our Voice citizen scientists collect information about their local community environment, work together to set priorities for action and create actionable solutions to improve their environment.

The Our Voice framework builds on the tenets of CBPR, and adds additional dimensions involving the following activities: community members collect information facilitated by Our Voice-specific mobile technology (i.e., a mobile app, described below), and then residents receive guidance and training to enable them to build neighborhood consensus, prioritize their results, frame recommended solutions to the issues that they identify, and engage directly with decision-makers to make changes in their local environments aimed at promoting positive health behaviors (e.g., improving side walk quality to promote more walking, developing back yard gardens to promote healthier eating).

**Fig. 1:**
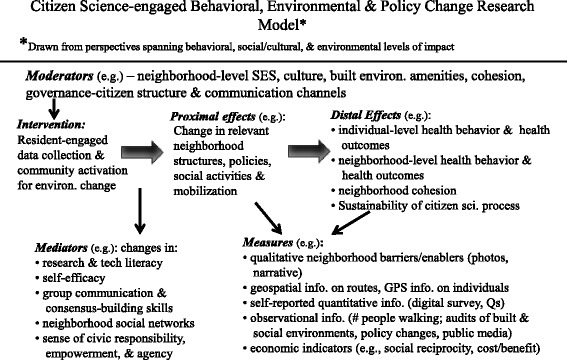
Citizen science-engaged behavioral, environmental, and policy change research model as adopted from King, 2015 [17]

While there are decades of research supporting the associations between a range of built and social environmental factors and higher levels of physical activity, healthy food access, and other positive health-related outcomes, few systematic approaches exist for engaging local residents as activators of healthy local environmental changes. The Our Voice Framework (Fig. 1) represents a type of trans-directional ecological model in which reciprocal person by environment interactions are explicitly targeted as a means of creating impacts at multiple levels of influence (i.e., individual, built and social environments, policy) [17]. The goal of such models is to actively traverse levels of impact through using agents at one level of influence (individual residents) to activate change at other levels of influence (e.g., the built environment, policy makers). Rather than focusing primarily on physical activity change at the individual level, citizen scientists in the Our Voice framework learn how to change elements of their local environments to enhance healthy and active lifestyles for everyone engaging with those environments. The solutions they develop and implement often involve activating those in decision making roles who can facilitate health-promoting environmental and policy level changes [17]. For example, in one application of the Our Voice framework, ethnic minority older adults living in a low-income community in the U.S. were able to work effectively, over a two-year period, with their city’s planning and engineering divisions and other local organizations to help facilitate the implementation of a community sidewalk inventory and repair program, a streetscapes and pathways review around their senior housing site to promote safer walking, improved access to the local senior centre, and the development of a community garden located adjacent to the senior housing site [65, 66].

In an Our Voice project in Cuernavaca, Mexico called Nuestra Voz, adolescent and adult residents living in low-income neighbourhoods were able to successfully use the Our Voice Discovery Tool to identify those local aspects that hindered residents’ ability to walk safely in their neighbourhoods. Because access to local decision makers and policy makers in their area was limited, the residents were able to agree on social environmental solutions, including creation of a neighbourhood residents committee aimed at better control of dogs in the neighbourhood (i.e., more frequent leashing and clean-up after dogs), a neighbourhood watch program to combat local crime, and strategies to enhance neighbourhood social cohesion [67].

A third example of a successful Our Voice project was aimed at improving access to healthy food choices in ethnically diverse older adults (including those of Latino, Chinese, and European descent) living in low-income neighbourhoods in northern San Mateo County, CA. The residents used the Discovery Tool to identify aspects of their local environments that helped or hindered healthy eating, and learned how to advocate for changes in partnerships with local decision and policy makers [68]. Participants learned about the food assistance and transportation services in their area about which most were previously unaware (e.g., regular food distribution programs, the US Department of Agriculture’s Supplemental Nutrition Assistance Program eligibility, congregate meal programs, existing and planned bus routes and shuttle services, financial assistance). At three months post-intervention, 84% of participants had contacted a local decision maker and/or shared information and resources with family, neighbours, or friends [68]. At 6 months post-intervention, older adult participants living in an affordable senior housing site in the targeted locale formed a Senior Advocacy Team (SAT), which met regularly to discuss and address issues of relevance to the older adults living at the housing site. Among the successful outcomes generated by the SAT was the hosting of an open forum at the housing site in which local city and county policy and decision makers discussed local environmental concerns and brainstormed solutions. Environmental changes that came out of this forum included modifications to street signage and curb designations which made it easier for the older adults to observe traffic and cross the street safely [68]. Post-intervention 12- and 24-month evaluations indicated that SAT members had attended a senior advocacy event twice in the California state capital, where they spoke with their state legislators concerning increased funding for affordable and safe senior housing [68]. They also partnered with an elementary school adjacent to the housing site to improve local traffic safety concerns, which resulted in an assessment of local traffic density and speed by the local transportation and housing departments. The results from this assessment led to subsequent environmental modifications by local government to enhance the nearby crosswalk and install pedestrian signal lights [68].

The Our Voice mobile technology, called the Stanford Healthy Neighborhood Discovery Tool (Discovery Tool), is an easy-to-use mobile device-based application that was developed by a team of research and community practice personnel from the Stanford Prevention Research Center and the broader Stanford community to assist residents in identifying neighborhood features that affect active and healthy living. The Discovery Tool has subsequently undergone extensive refinement based on input from scientists and community users in the U.S. and globally. The Discovery Tool records GPS-tracked walking routes, and allows residents to capture geo-coded photographs and audio narratives which identify barriers to and enablers of healthy living in their neighborhoods (e.g., healthy food access, features that affect neighborhood walkability; health food access) [69]. The Discovery Tool was originally developed using a CBPR approach and enables users to capture, in a standardized way, relevant contextual data about their local environments in real time, which facilitates later data analysis and community decision-making. The Discovery Tool has been used thus far in a growing number of studies across three continents. These studies have provided evidence for its ease of use and acceptability among a range of users [12]. For example, low-income, technology-naïve Latino adolescents and older adults used it in a pilot study conducted in the North Fair Oaks neighborhood of Redwood City, CA to assess neighborhood built environment features that helped or hindered their physical activity [70]. In addition to the U.S., the Discovery Tool has been used by residents living in Mexico, Colombia, Chile, and Israel [12]. The tool was shown to be an easy-to-use technology-driven tool that has facilitated citizen science research and collaborations [12].

In the five years since the first publication describing the Discovery Tool and the Our Voice framework, additional projects have been planned or initiated in several regions of the United States, Canada, the United Kingdom, Australia, Brazil, Germany, New Zealand and South Africa [12]. The Our Voice Global Network (http://med.stanford.edu/ourvoice/the-global-network-right.html) was formed to create a learning community around the Our Voice model and coordinate approaches across geographically disparate projects.

## Discussion

The purpose of this paper is to provide recommendations to researchers utilizing Citizen Science participatory approach for measures that should be considered for inclusion in future citizen science participatory projects addressing physical activity, and to offer some examples of specific instruments available to conduct these assessments. Encouraging consistency across studies can accelerate the efficiency with which citizen science participatory research provides new insights and solutions to behaviorally-based public health issues that drive morbidity and mortality around the world [2].

We have elaborated on one specific example of the citizen-engaged process, referred to as the Our Voice framework. Since its inception in 2012, the Our Voice framework has been implemented in several countries. This momentum has created an opportunity for the development and adoption of shared research approaches to allow comparison between the Our Voice approach and similar citizen science activities in a wide range of settings. The adoption of recommendations reported in this paper would create the potential for synergies among future Our Voice projects and similar projects.

The measures described in this paper abide by four principles selected for inclusion in citizen science participatory projects, as follows: feasibility, utility, propriety, and accuracy. The choice of measures will take into account the resources available for outcome and process evaluation. We stress the importance for all such projects to follow an evidence-based approach and ensure that they incorporate an appropriate assessment protocols. Not only will such an approach magnify the impact of individual projects, but it will also highlight the shared strengths and opportunities for further development of these projects leading to the creation of a critical mass of well-coordinated studies. This is an exceptional opportunity to further the community-based health promotion agenda which in the past has suffered from a lack of coordination, shared vision and comparable research measures.

The strengths of this paper include the following: i) the constructs and measures align with citizen science participatory projects and the Our Voice framework; ii) it provides a guide for researchers embarking on community-engaged research; iii) it focuses on variables that are often ignored (e.g., civic engagement; sense of empowerment); and iv) it includes both quantitative and qualitative measures. A limitation to the information provided is the omission of specific and authoritative recommendations as to which measures should be employed for which studies, and the absence of a thorough review of all available tools for assessing each construct. This level of detail is both beyond the scope of this paper and impractical, given the many and varied factors that may influence the choice of measures. These factors may include available resources, characteristics of the community residents, expertise within the study team and the community, and the specific target of the program or intervention. Moreover, this paper focuses primarily on constructs relevant to promoting physical activity, whereas citizen science participatory projects can be used to address many issues, including nutrition (e.g., access to healthful food options), recycling (e.g., ease of neighborhood recycling), and access to health care. Many of the constructs identified in this paper may be relevant to these other arenas, but each of these target areas will require attention to additional constructs that may not have been mentioned here. This area is in a relatively early stage of research and a growing body of evidence would be required for recommendations to be presented. This paper highlights important areas to consider in order to ensure collection of high quality evidence which will move this field forward.

Among the challenges of citizen science participatory research is capturing longer-term changes (i.e., those occurring well beyond the initial citizen scientist engagement process). Researchers are not usually funded for more than two to three years, or even shorter time periods. The impact on the built and social environments may only come to fruition in the longer term. Thus, it becomes important to assess the shorter-term individual and environmental changes that may serve as markers or facilitators of longer-term environmental or policy impacts in a locale. Such markers include the natural diffusion of citizen scientist activities to other local topic areas of concern or to other groups of residents, as has been observed in the few longer-term Our Voice projects to date [66, 68]. Identifying funding opportunities that support the longer-term follow-up of such community-based citizen science projects is also essential in helping to bridge the gap between evidence and practice in this field. Another challenge is capturing changes at multiple levels of impact, from the individual through community levels. One way of resolving this challenge may be by aggregating individual responses to the neighborhood level and analyzing this information as an ecologic rather than an individual characteristic [71].

## Future directions and next steps

Advances in technology provide the possibility of enriching citizen science participatory research through combining them with new or novel sources of data. Some examples include the following:

- virtual or augmented reality to show policy makers, community partners, and residents alike how proposed changes would actually look and “feel”. The display of such information could enable more confident decision making and more thorough consideration of all possible alternatives, thereby enhancing the decision making process [72].
- the use of existing closed-circuit television (CCTV) cameras (Archive of Many Outdoor Spaces. http://amos.cse.wustl.edu/), where a collection of long-term time-lapse imagery from publicly accessible outdoor webcams around the world are used.
- linking Our Voice data to other types of aggregated health data for a particular locale, such as health insurance or public health data records.
- incorporating geographic information systems (GIS) data, local census data, and wearable activity monitors and other biometric sensors (e.g., FitBit, Apple Watch, stress sensors, etc.) with Discovery Tool and similar resident-generated data.
- Including measures that are used in health impact assessments.
- The use of online tools (e.g. Smartsheet) to track, manage and automate collaborative projects.

## Conclusion

The citizen science participatory approach to research embodied in the Our Voice framework represents a paradigm shift in the basic conceptualization of population health science as currently practiced. This paradigm departs from the traditional approach in which trained scientists, often residing outside of the communities or populations which they are studying, are the drivers of community question generation, data collection, and data interpretation. The citizen science model facilitates scientific partnerships with engaged residents through helping them systematically apply a replicable set of tools and processes to improve the health of the local communities in which they live. The role of researchers is to complement the deep knowledge that citizen scientists have about the issues facing their communities with knowledge about the scientific process. Providing citizen scientists with training (for example, how to gather, review and analyze data), and resources, (such as workbooks, manuals, and templates), can facilitate the development of systematized, replicable processes. Building and sustaining mutual trust is key to overcoming the challenges of conducting citizen science research [73]. These challenges include ensuring cultural appropriateness, recruiting and retaining citizen scientists as participants, building academic-community partnerships, resolving conflicts, ensuring momentum and productivity, and promoting long-term sustainability [73].

In maintaining and enhancing Citizen Science engagement, we highly recommend that the guiding principles of community based participatory research are followed, which include the following activities: recognizing the community as a unit of identity; building on strengths and resources within the community; facilitating collaborative partnerships through open, transparent, and respectful interchanges and discussion throughout the project; integrating knowledge and action for the mutual benefit of all partners; promoting a co-learning and empowering process that attends to social inequalities; using a cyclical and iterative process to ensure that interventions are optimized for the target population or community; addressing health from both positive and ecological (contextual) perspectives; and disseminating the findings and knowledge gained to all partners in ways that are readily understood [74]. We believe that the potential of these approaches and suggestions in fostering contextually meaningful physical activity-enabling solutions is high and worthy of further exploration and development.
